# Extent of linkage disequilibrium and effective population size of Korean Yorkshire swine

**DOI:** 10.5713/ajas.17.0258

**Published:** 2018-07-26

**Authors:** Donghyun Shin, Kyeong-Hye Won, Sung-Hoon Kim, Yong-Min Kim

**Affiliations:** 1 Department of Animal Biotechnology, Chonbuk National University, Jeonju, 54896, Korea; 2 PigGene Korea Inc., Yongin 16866, Korea; 3 Korean Bioinformation Center, Korea Research Institute of Bioscience and Biotechnology, Daejeon 34141, Korea

**Keywords:** Linkage Disequilibrium, Yorkshire, Effective Population Size, Single Nucleotide Polymorphism

## Abstract

**Objective:**

We aimed to characterize linkage disequilibrium (LD) and effective population size (N_e_) in a Korean Yorkshire population using genomic data from thousands of individuals.

**Methods:**

We genotyped 2,470 Yorkshire individuals from four major Grand-Grand-Parent farms in Korea using the Illumina PorcineSNP60 version2 BeadChip, which covers >61,565 single nucleotide polymorphisms (SNPs) located across all chromosomes and mitochondria. We estimated the expected LD and inferred current N_e_ as well as ancestral N_e_.

**Results:**

We identified 61,565 SNP from autosomes, mitochondria, and sex chromosomes and characterized the LD of the Yorkshire population, which was relatively high between closely linked markers (>0.55 at 50 kb) and declined with increasing genetic distance. The current N_e_ of this Korean Yorkshire population was 122.87 (106.90; 138.84), while the historical N_e_ of Yorkshire pigs suggests that the ancestor N_e_ has decreased by 99.6% over the last 10,000 generations.

**Conclusion:**

To maintain genetic diversity of a domesticated animal population, we must carefully consider appropriate breed management methods to avoid inbreeding. Although attenuated selection can affect short-term genetic gain, it is essential for maintaining the long-term genetic variability of the Korean Yorkshire population. Continuous and long-term monitoring would also be needed to maintain the pig population to avoid an unintended reduction of N_e_. The best way to preserve a sustainable population is to maintain a sufficient N_e_.

## INTRODUCTION

Several important evolutionary processes in finite populations, including migration, artificial or natural selection, and genetic drift lead to non-random association of alleles between two different loci, or linkage disequilibrium (LD) [1]. Recent genomic methods in animal breeding, such as genome-wide association studies (GWAS) and genomic selection using single nucleotide polymorphism (SNP) data, depend on the extent of LD and its rate of decline with distance between loci of the population. Researchers have applied SNP chips for GWAS [2–4] and genomic selection studies [5–7] in pigs but have found that most traits of interest are complex and more suitable for genomic selection techniques than GWAS for studying significant associations in some genomic regions [8]. Methods used to study animal breeding genetics rely strongly on the quality of LD and sample size. Therefore, characterization of LD is very important for planning future genomic studies of complex traits relevant to animal breeding.

The LD among loci can provide insights into the evolutionary history of each population by using effective population size (N_e_), where N_e_ is the number of individuals in an idealized population that would yield the same inbreeding degree as the real population [9]. Thus, we can monitor genetic diversity in each domesticated animal population based on N_e_ and explain the observed extent and pattern of genetic variation in population genetic terms. Using N_e_, we can also predict loss of genetic variation from a prospective point of view and the accuracy of natural genomic selection before the emergence of artificial genomic selection in domesticated animal breeding. Additionally, we can infer ancestral N_e_ using the strength of LD at different genetic distances between markers. The pattern of historical N_e_ in each animal population can increase our understanding of the impact of recent strong artificial selection breeding methods on population-level genetic variation. If pedigrees are incomplete or unavailable, we can use inbreeding information of populations of interest with respect to N_e_.

In Korea, several pig breeds are economically significant, including Yorkshire, Landrace, Duroc, and Berkshire, though Yorkshire is one of the most important breeds because has an excellent maternal line index and outnumbers all other maternal line pig breeds in Korean Grand-Grand-Parent (GGP) farms. Patterns of LD in Yorkshire populations in other countries have been characterized previously, and their N_e_ were predicted using SNP chip data. For example, the extent of LD of multiple pig breeds, including Yorkshire, from the United States, Denmark, and The Netherlands were investigated [10–12]. In addition, Uimari determined the extent of LD and estimated LD-based actual N_e_ and ancestral N_e_ of 32 Finnish Yorkshire boars [13]. Although the Korean pig industry is active, Korea has no original Yorkshire breed and relies on significant pig imports. As a result, the Korean Yorkshire population has diverse genetic sources from several countries, so N_e_ could be an important measurement of both the Korean and global pig industries.

In our current study, LD and N_e_ in a Korean Yorkshire population were estimated using data generated with the Illumina PorcineSNP60 v2 BeadChip (Illumina Inc., San Diego, CA, USA). We also investigated the ancestral N_e_ of the population. Together with findings from other studies, our results can inform the establishment and implementation of the most effective animal breeding genomic methods for Korean Yorkshire swine.

## MATERIALS AND METHODS

### Study sampling and selection of genotypic data

This study included genotypic data from 2,470 Yorkshire pigs from four representative major GGP farms (1,473 from Farm 1, 542 from Farm 2, 312 from Farm 3, and 143 from Farm 4) in Korean, constituting a representative sample of the Korean Yorkshire population. All pigs were pigs from each major GGP farms in 2015 through 2016 (Supplementary Figure S1). The Illumina PorcineSNP60 v2 BeadChip (Illumina Inc., USA), which contains 61,565 SNPs located across all autosomes, mitochondria, and sex chromosomes, was used in this study. These informative SNPs were selected from the recent porcine reference genome (Build 9) from the Ensembl database. The generated genotypic data were analyzed using PLINK (version 1.90) for quality control. First, we extracted 52,257 autosomal SNPs from the total 61,565 SNPs on all autosomes and then removed certain SNPs from analysis due to poor genotyping qualify; 9,303 SNPs were excluded based on Hardy-Weinberg equilibrium testing (p≤0.000001). The total genotyping rate of remaining sequences was 0.996, but 480 SNPs failed the missingness test (missing genotype>0.1), and 10,160 SNPs with a minor allele frequency (MAF) of <0.05 were excluded. The criteria for genotyping quality used were similar to those described previously [13] and yielded 33,418 SNPs for downstream analysis. We performed imputation to replace missing data with substituted genotypes [14] before LD and N_e_ were characterized.

### Characterization of linkage disequilibrium in Korean Yorkshire pigs

We estimated LD using the LDcorSV R package (version 1.3.1). Pairwise haplotype frequencies were estimated from the genotype data and used to calculate the squared correlation coefficient between two loci (*r*^2^). The *r*^2^ are respectively equivalent to the covariance and correlation between alleles at two different loci computed as:

(1) $$r^{2}=\frac{D^{2}}{P_{A}P_{a}P_{B}P_{b}}$$

Where *P_A_*, *P_a_*, *P_B_*, and *P_b_* are the respective frequencies of alleles *A*, *a*, *B*, and *b*, and *D* is *P_AB_* - *P_A_P_B_*.

**Figure 1:**
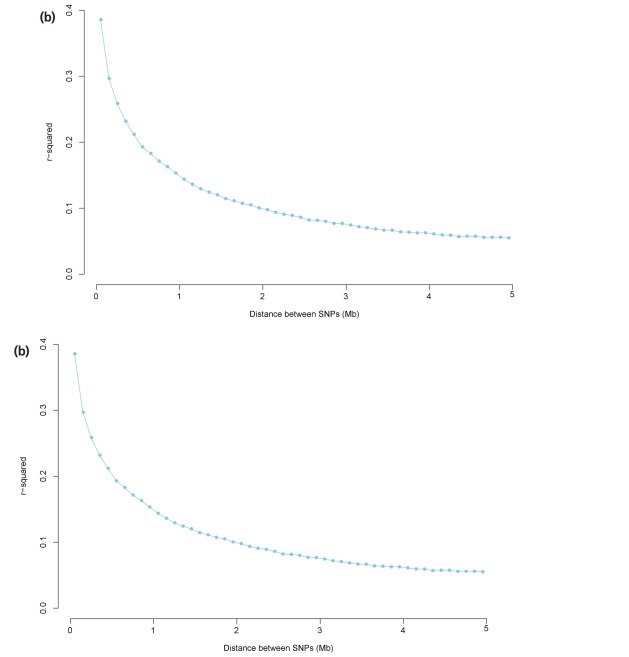
Average linkage disequilibrium (LD) plotted against the median of the distance bin range (Mb). (a) Distances ranged from 0 to 0.5 Mb, and *r*^2^ values were averaged using bins of 0.01 Mb and pooled over autosomes. (b) Distances ranged from 0 to 5 Mb, and *r*^2^ values were averaged using bins of 0.1 Mb and pooled over autosomes.

For each SNP, pairwise LD was calculated for its adjacent 100 SNPs. The SNP quality and distance requirements resulted in approximately 3.2 million SNP pairs distributed over the total genome. This *r*^2^ calculation was performed on a chromosome-by-chromosome basis and illustrated a relationship of physical distance and *r*^2^ between the two target loci per chromosome (Supplementary Figure S2). Details of the SNPs’ physical position can be found in the Illumina product literature. To determine LD in relation to the physical distance between SNPs, SNP pairs were divided into distance bins. We established two classes (0 to 0.5 Mb and 0 to 5 Mb), and applicable SNP pairs in each class were placed into 1 of 50 distance bins with class-dependent bin ranges (Supplementary Table S1). The two types of mean *r*^2^ for each distance bin were plotted against the median of the distance bin range, which are presented in Figure 1.

### Construction model of linkage disequilibrium with distance

Under the assumption of an isolated population with random mating, Sved derived an approximate expression for the expectation of *r*^2^ [15]:

(2) $$E(r^{2})=\frac{1}{1+4Nc}$$

Where *N* is N_e_, and *c* is the recombination frequency. We replaced *c* with linkage distance in morgans [16–18], and our calculations were further supported by approximating the more precise equation for *E*(*r*^2^) given by Sved [15]. Based on this formula, a non-linear least-squares approach to statistically model the observed *r*^2^ was implemented within R using this model:

(3) $$y_{i}=\frac{1}{a+4bd_{i}}+e_{i}$$

Where *y_i_* is the value of *r*^2^ for the SNP pair *i* at linkage distance *d_i_* in morgans. Parameters *a* and *b* were estimated iteratively using the least-squares method. Chromosome-specific Mb-to-centimorgan (cM) conversion rates were calculated based on total physical chromosome length stated on the UCSC Web site (https://genome.ucsc.edu/) and each chromosome’s length from a porcine linkage map from maps of four pedigrees (ILL, UIUC, USDA, ROS) [19]. Because UIUC consisted of Meishan and Yorkshire, we selected maps of this pedigree for use in our study. This model was applied to data for each chromosome and estimated parameter. Similar to Corbin and Shin, we combined the estimated parameters into a meta-analysis using an inverse variance method for pooling and a random-effects approach based on the DerSimonian-Laird method [18,20].

### Ancestral N_e_ estimation

Equation (2) can predict the N_e_ at a given point in time expressed as the past generation [16]:

(4) $$N_{T}(\text{t})=\frac{1}{4c}*\left(\frac{1}{r_{c}^{2}}-1\right)$$

Where *N_T_(t)* is the N_e_ at *t* generations ago, *c* is the distance between markers in morgans, $r_{c}^{2}$ is the mean value of *r*^2^ for SNP pairs *c* morgans apart, and *c* = 1/2*t* when assuming linear growth [16]. To estimate *N_T_(t)*, the number of prior generations was selected, and a suitable range for *c* was calculated. The binning process was designed to ensure sufficient SNP pairs within each bin to obtain a representative *r*^2^ mean. This process was performed for SNPs pooled across autosomes. Bin information used for estimating ancestral N_e_ is presented in Supplementary Table S3.

## RESULTS

### Characteristics of genotypic data

**Figure 2:**
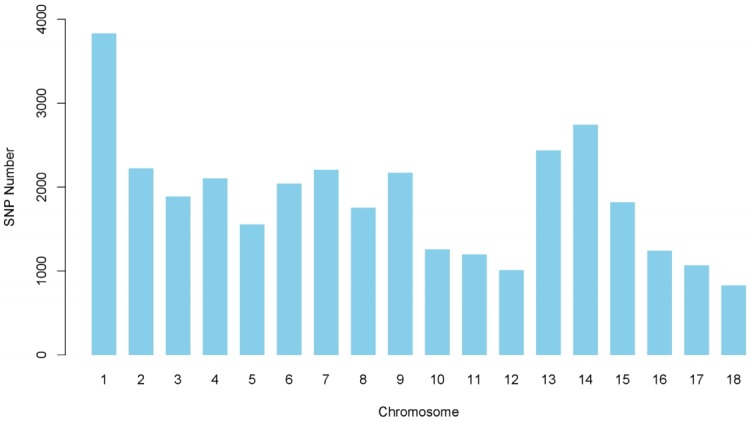
Number of single nucleotide polymorphisms (SNPs) per chromosome after quality control processing and imputation for estimation of effective population size. The *x*- and *y*-axes indicate SNP numbers and chromosomes, respectively.

To investigate genetic variations in 2,470 Yorkshire pigs from four representative major Korean major GPP farms, chip analysis was performed on 33,418 SNPs after quality control processing. The number of SNPs per autosome remaining after filtering and imputation ranged from 831 to 3,834 and was closely related to chromosome length and total number of SNPs (Figure 2). The MAFs of remaining SNPs followed a uniform distribution and averaged (±standard deviation [SD]) 0.287±0.127. The average distance between SNP pairs (±SD) was 3,610±2,831 kb, with the distance between SNPs ranging from 0.009 kb to 24,564 kb (Supplementary Figure S3).

### Estimation of linkage disequilibrium

The average LD (*r*^2^) of 3.2 million SNP pairs (±SD) analyzed in this study was 0.103 (±0.179). Distance of 28,969 pairs from all studied pairs was less than 50 kb. Overall, 51.22% of these 28,969 SNP pairs had *r*^2^>0.3, and 60.75% had *r*^2^>0.2. The average LD of distinct autosomes for SNPs at least 50 kb apart varied from *r*^2^ = 0.361 to *r*^2^ = 0.508, and the average LD for SNPs at least 5 Mb apart ranged from *r*^2^ = 0.071 to *r*^2^ = 0.185 (Supplementary Table S4). Although the aim of this study was not to compare LD in different chromosomes, we did observe some variation in the extent of LD by chromosome. We also determined that chromosomes 1, 13, and 14 had the highest average LD values, while chromosomes 10 and 12 showed the lowest average LD values. These results corroborate findings from a previous Yorkshire LD study.

Average LD values across all autosomes showed that the most rapid decrease was seen over the first ten bins (distance between SNPs ranged from 0 to 0.1Mb), with the mean *r*^2^ decreasing by approximately 40% (Figure 1a). The most rapid decrease was seen over the first five bins with the mean *r*^2^ decreasing by approximately 50% (Figure 1b). Mean *r*^2^ decreased much more slowly as distanced increased and was constant after 3 Mb of distance. According to our *r*^2^ calculations, 3,965 of the 3.2 million SNP pairs were in complete LD.

### Determination of the relationship between linkage disequilibrium and single nucleotide polymorphism distance

**Figure 3:**
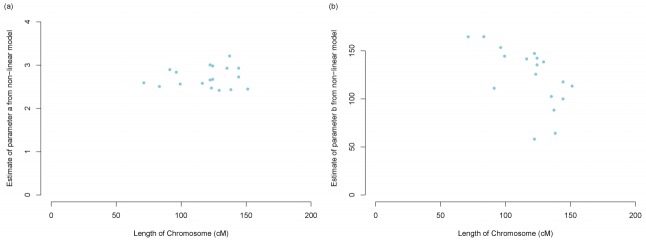
Parameter estimates from Equation (3) plotted against chromosome length in centimorgans (cM) according to the referenced porcine linkage map *r*^2^ used in our study. (a) Estimates of parameter *a* in Equation (3) plotted against chromosome length (cM). (b) Estimates of parameter *b* in Equation (3) plotted against chromosome length (cM).

**Figure 4:**
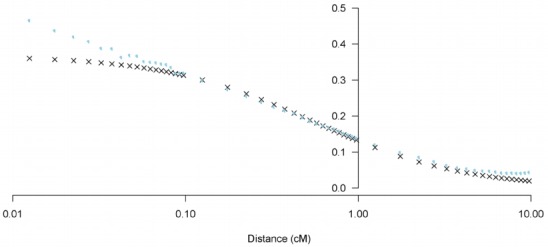
Predicted *r*^2^ (Black x) vs observed *r*^2^ (sky blue circle) against mean distance between markers (cM expressed on a log scale). Predicted *r*^2^ calculated using Equation (3) with a = 2.71 and b = 122.87.

Application of the non-linear regression model of declining LD in accordance with distance determined both parameters *a* and *b* in Equation (2) are significantly different from zero. For parameter estimation using *r*^2^, the mean estimate and 95% confidence interval by meta-analysis across autosomes for parameters *a* and *b* (±SD) were 2.71 (2.61; 2.82) and 122.87 (106.90; 138.84), respectively. Values for estimated parameters *a* and *b* in Equation (2) per chromosome are shown in Figure 3. Parameter *b* showed greater variability between chromosomes than parameter *a*. No such relationship was observed between each estimated parameter and chromosome length in centimorgans. We estimated predicted r2 by distance of SNP pairs using our estimated parameters *a* and *b* in Equation (2) and compared predicted *r*^2^ with observed *r*^2^ as performed in other studies [17,18]. We determined that the predicted *r*^2^ from the non-linear regression equation was similar to the mean observed *r*^2^ (Figure 4), indicating our estimated parameters in Equation (2) accurately represent Korean Yorkshire population history.

### Ancestral N_e_ estimation

**Figure 5:**
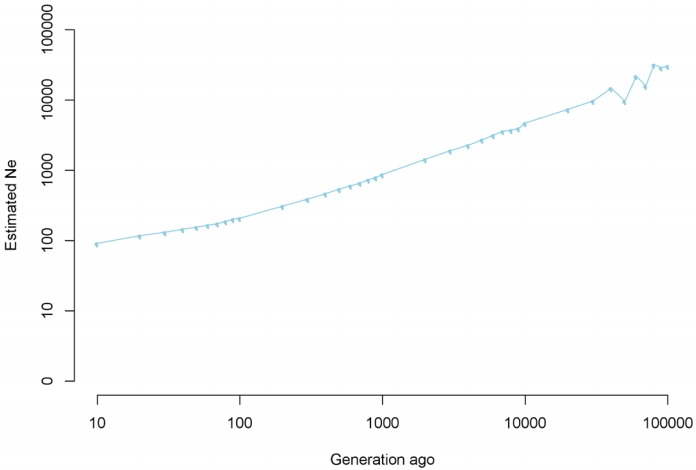
Average estimated N_e_ (effective population size) plotted against number of past generations truncated at 100,000 generations using linkage disequilibrium (*r*^2^) plotted on a log scale.

Figure 5 and Supplementary Figure S4 display estimated N_e_ at *t* generations ago. Based on the genomic data, the current N_e_ of Korean Yorkshire is approximately 122.87 (106.90; 138.84) individuals. Supplementary Figure S4 also shows that the reduction of N_e_ in Korean Yorkshire populations was continuous and gradual over the last 100 generations, ranging from 208.4 individuals to 122.87 individuals as determined in our study. Additionally, we found that Korean Yorkshire N_e_ has decreased by 99.6% over the last 10,000 generations (30,380.28 initial individuals) to the present estimate (Figure 5).

## DISCUSSION

**Figure 6:**
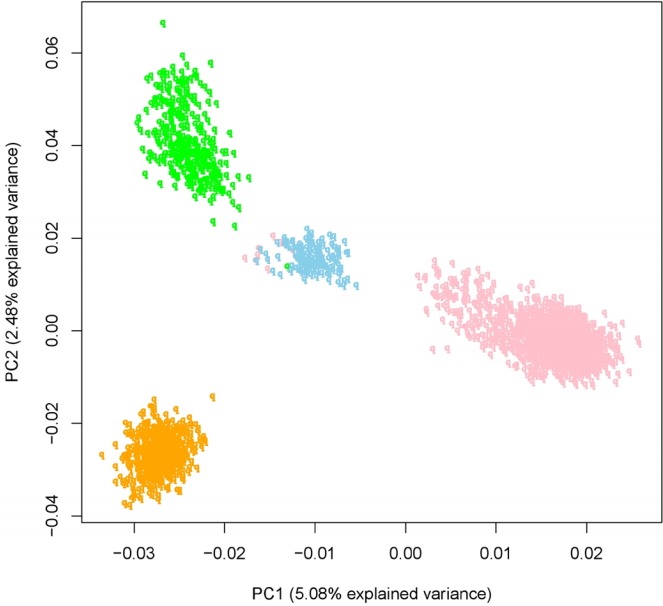
PCA (Principal component analysis) plot of Yorkshire population (2,470 Individuals) in this study. The 2,470 individuals Yorkshire population belonged to 4 GGPs and each color indicated each farms.

Four GGP farms in this study were operated by each different farm owners, but they were connected by sharing some semen. To show the genetic background of these population, we made principal component analysis plot including PC1 and PC2 (Figure 6). We could found that four groups were clearly distinguished, but the difference is not large, and the explanation variance of PC1 and PC2 is also very small. So we regarded four representative GGP farms as one Yorkshire population in Korea. We removed 9,303 SNPs by the quality control of Hardy-Weinberg equilibrium test for 2,470 Yorkshire pigs. We thought that the reason why so many SNPs removed by the quality control was related to number of heterozygous SNP alleles. A strong degree of selection in GGP would have reduced the effective population and had an impact on the number of heterozygous alleles, which could increase the degree of loss of heterozygosity. For this reason, we thought that many SNPs have been removed. Additionally, becasue core objective was estimation of N_e_ of Yorkshire population in Korea, we need one Yorkshire population in Korea and high quality SNPs of this population. And we thought that number of SNPs (33,418 SNPs) after quality control was sufficient for estimation of N_e_ when compared to finnish Yorkshire (Uimari [13]). So we thought that regarding 4 GGP farms as one Korean population certainly was appropriate.

We investigated the extent of LD and changes of the N_e_ of the Korean Yorkshire population based on whole-genome SNP data. The observed LD, measured as *r*^2^, extended for a long distance based on the adjacent 100 SNPs of each SNP studied in the genome. A previous study used large pedigree datasets and small genomic datasets [13], but instead of pedigree data, we used large-scale genomic data from swine in GGP farms to characterize LD and estimate N_e_. Because domesticated pigs have long been strongly and artificially selected, observed LD in the Korean Yorkshire population was higher with shorter genomic distances and more extensive compared to human populations [21]. Declining LD in the Korean Yorkshire population is consistent with previous studied pigs [13,22] as well as other domesticated animals [17,18].

We estimated N_e_ of Korean Yorkshire swine based on a non-linear regression model that describes the relationship between linkage distance and LD. Estimating N_e_ using this equation raises difficulties in handling values in the limits of the parameter space, because if *r*^2^ = 0.0, the estimated N_e_ is infinite, and if *r*^2^ = 1.0, N_e_ is zero. In this study, we calculated *r*^2^ for the adjacent 100 SNPs of each SNP to decrease bias in handling these values. The results from this simplified approach yielded quite similar estimates of N_e_ as other previous studies. Another concern related to the relationship between estimated LD and distance between SNPs lies in the accuracy of the porcine reference genome used in this study. The order and distance between SNPs in the commercial Illumina PorcineSNP60 v2 BeadChip will likely be refined because the reference genome version will also be updated. However, the bias from incorrectly ordered SNPs or wrong SNP distances between SNPs may be minimized by the large number of SNP pairs used in this study, so some overestimated or underestimated LD can be overlooked. Because the relationship between genetic and physical distances varies across chromosomes and chromosomal regions, we inferred the Mb/cM ratio per chromosome using physical map position information from the porcine reference genome and from a previous Yorkshire pig study [19]. We then used genetic distances based on physical distances to estimate N_e_ and could obtain more reliable N_e_ estimates with such detailed estimates of genetic distances between SNPs. Finally, one study reported that a limited sample size can bias the estimates of *r*^2^ and recommends correcting the estimates of *r*^2^ for sample size *n* (*r*^2^–1/2*n*) and using the equation of Sved [15]. However, our sample size was sufficient enough to correct estimates of *r*^2^. To estimate N_e_ of Korean Yorkshire pigs, we used alternative version of this equation further derived by Tenesa [23], which adds a new parameter *a* to account for mutations. Based on the new formula, the initial value of parameter *a* = 2 in estimations of parameters using a non-linear regression model in R. Regarding variance heterogeneity of the observed *r*^2^, the variance declined with increased distances between SNPs, which may have impacted our results when estimating parameter *b* in Equation (2) (Supplementary Figure S2). A significant, negative relationship between chromosome length and estimates of parameter *b* obtained from a non-linear model have been observed [17], while others have noted a positive relationship in domestic livestock species [24,25] or did not investigate directionality of the relationship [18]. Because the evolutionary history of each species and breed is different, the relationship between chromosome length and parameter *b* is also different per population. In this study, all marker pairs were calculated in each bin so that *r*^2^ was not affected by chromosome length. These results are in agreement with the Yorkshire LD characterization findings of Uimari [13]. We did not observe a significant relationship between chromosome length and estimates of *b* in our study population.

Our estimate of *b* represents an estimated N_e_ assuming a constant present population size, because we used genetic data from the Korean Yorkshire population consisting of pigs in major GGP farms. In the calculation of N_e_, *b* in Equation (2) represents a conceptual average of N_e_ over the period inferred from the SNP pair distance ranges per chromosome [26]. We regarded parameter *b* combined by meta-analysis as reflecting the current N_e_ of the Korean Yorkshire population.

As Table 1 showed, we produced SNP chip data of 2,470 individuals in between 2011 and 2015. And approximately 87% (2,149 individuals) of total SNP data was produced in between 2014 and 2015. Because sampling period was short, we regarded sampled individuals in this study as “current generation population” in Korean Yorksrhie population. So we thought that we need not to sort 2,470 individuals data according to accurate generation. Instead, we infered long time generation-related change of N_e_ using another method in this study. Hayes [16] reported that the degree of linkage equilibrium according to genetic distance had reflected genetic diversity of past generation. After *r*^2^ estimation using SNP data, we divided *r*^2^ by distance and inferred N_e_ of past generation. The LD over greater genetic distances reflects a population’s recent history, whereas LD over shorter distances depends on the N_e_ many generations ago [16,27] (Supplementary Table 3). Historical N_e_ estimation suggests a linear population as reported in a previous study (Figure 5; Supplementary Figure S4) [16]. The observed pattern displayed a consistent decrease in N_e_ from 100 generations ago to the present, decreasing by 99.6% from 10,000 to 100 generation ago. Several explanations exist for this pattern, including bottlenecks associated with domestication, selection, and breed administration, business strategy, and endangerment of the breed. Therefore, our results should be considered in context of the demographic history of the Yorkshire population in Korea. The reliability of predicting changes in N_e_ over time depends both on technical implementation and proper iteration based on previous studies using this approach [17,18].

We aimed to characterize LD and N_e_ in a Korean Yorkshire population using genomic data from thousands of individuals. Our observed LD patterns are similar to the average value presented by Du for six commercial pig lines and Uimari for Finnish Yorkshire pig breeds [13,22]. The overall LD in Finnish Yorkshire breed appears to be stronger than in Korean Yorkshire pigs. Because the Korean Yorkshire population consists of seed pigs from several original Yorkshire breeds, the genetic diversity of Korean Yorkshire pigs is greater than that of the single Finnish Yorkshire breed, and the LD of Finnish Yorkshire pigs is higher than that of Korean Yorkshires.

The minimum number of breeding animals recommended by the UN Food and Agriculture Organization is 50, although Meuwissen suggested this number is the lower limit for a critical population size, proposing that the actual size should be between 50 and 100 [28]. The current N_e_ of the Korean Yorkshire population is 122.87, which is sufficient to maintain the population’s viability. The population’s genetic variation enables an acceptable inbreeding rate, including compromising genetic gain in commercially important traits. This genetic variation is necessary to apply methods that maximize selection efficacy with a fixed rate of inbreeding or optimize the use of genetic resources from the parental generation [29].

When we apply a new genetic method, such as genomic selection, for estimating breeding values, the N_e_ may be very small or continually decrease [8]. Therefore, one must carefully consider appropriate breed management methods to avoid inbreeding. Although attenuated selection can affect short-term genetic gain, it is essential for maintaining the long-term genetic variability of the Korean Yorkshire population. Long-term continuous monitoring would also be needed to maintain the pig population to avoid an unintended reduction of N_e_. The best way to preserve a sustainable population is to ensure sure its production populations maintain a sufficient N_e_.

## Supplementary Data



## Figures and Tables

**Table 1:** Sample information of Yorkshire population (2,470 Individuals) in this study

Year	Number of individual	Farm1	Farm2	Farm3	Farm4	Female	Male
2011	5	0	0	1	4	1	4
2012	137	4	0	59	74	134	3
2013	179	23	0	124	32	167	12
2014	687	122	430	109	26	674	13
2015	1,462	1,324	112	19	7	1,166	296
Total	2,470	1,473	542	312	143	2,142	328
