# Supplementary material for: Extent of linkage disequilibrium and effective population size of Korean Yorkshire swine
Source: Asian-Australas J Anim Sci. 2018 Jul 26;31(12):1843–51. doi: 10.5713/ajas.17.0258 (PMC6212734; doi:10.5713/ajas.17.0258)

Supplementary material

Title:

Extent of linkage disequilibrium and effective population size of *Yorkshire* population in Korean

Authors Information:

Donghyun Shin[1], Kyeong-Hye Won[1], Sung-Hoon-Kim[2], Yong-Min Kim[3,*]

[*] Corresponding author: Yong-Min Kim

Tel: +82-42-879-8534, Fax: +82-42-870-8519, E-mail: ymkim@kribb.re.kr

[1]Department of Animal Biotechnology, Chonbuk National University, Jeonju, 54896, Republic of Korea

[2]PigGene Korea Inc., Yongin, 16866, 3Sunjin Co. LTD, Seoul, 05372, Republic of Korea

[3]Korean Bioinformation Center, Korea Research Institute of Bioscience and Biotechnology, Daejeon, 34141, Republic of Korea

Supplementary Table 1. Distance classes and bin ranges for the linkage disequilibrium summary

Class	Minimum distance (Mb)	Maximum distance (Mb)	Within class bin distance range (Mb)	No of bins
1	0	0.5	0.01	50
2	0	5	0.1	50

Supplementary Table 2. Chromosome-specific centimorgan to megabase (cM/Mb) conversion ratios

Chromosome	Length (Mb)	Length (cM)	cM/Mb ratio
1	315	144	0.457
2	163	137	0.843
3	145	122	0.843
4	143	129	0.899
5	112	124	1.112
6	158	151	0.957
7	135	144	1.069
8	148	124	0.835
9	154	135	0.879
10	79	116	1.466
11	88	96	1.095
12	64	99	1.557
13	219	122	0.558
14	154	138	0.897
15	158	123	0.780
16	87	91	1.047
17	70	83	1.191
18	61	71	1.160
Total	2,453	2,149	-

Supplementary Table 3. Description of the generation binning process

Generation range applied to	Number of generations represented by each bin	Number of bins	Example for first bin		
			Generation	Generation range	Corresponding distance range (Morgans)
10 to 100	10	10	10	5 to 15	0.033 to 0.1
200 to 1,000	100	9	200	150 to 250	0.002 to 0.003
2,000 to 10,000	1,000	9	2,000	1,500 to 2,500	0.0002 to 0.0003
20,000 to 100,000	10,000	9	20,000	15,000 to 25,000	0.00002 to 0.00003

Supplementary Table 4. Average linkage disequilibrium (LD) in different autosomal chromosomes for SNP 50 kb and 5 Mb apart

Chromosome	Less than 50 kb between two SNPs		Less than 5Mb between two SNPs	
	n	r2	n	r2
1	3,099	0.480	261,355	0.163
2	2,067	0.379	165,375	0.119
3	1,566	0.390	128,153	0.094
4	1,808	0.419	164,486	0.103
5	1,312	0.393	111,216	0.083
6	1,926	0.445	140,143	0.112
7	1,932	0.378	178,958	0.096
8	1,449	0.401	116,303	0.095
9	2,029	0.430	161,040	0.111
10	1,136	0.373	100,056	0.071
11	942	0.384	85,855	0.080
12	1,059	0.361	77,304	0.071
13	1,844	0.508	157,021	0.185
14	2,525	0.471	241,198	0.158
15	1,513	0.470	120,914	0.116
16	1,081	0.379	90,613	0.098
17	994	0.379	83,330	0.077
18	687	0.418	56,632	0.076
Total	28,969	0.424	2,439,952	0.116

Supplementary Figure 1. Average estimated effective population size plotted against generations in the past, truncated at 100 generations using r2.

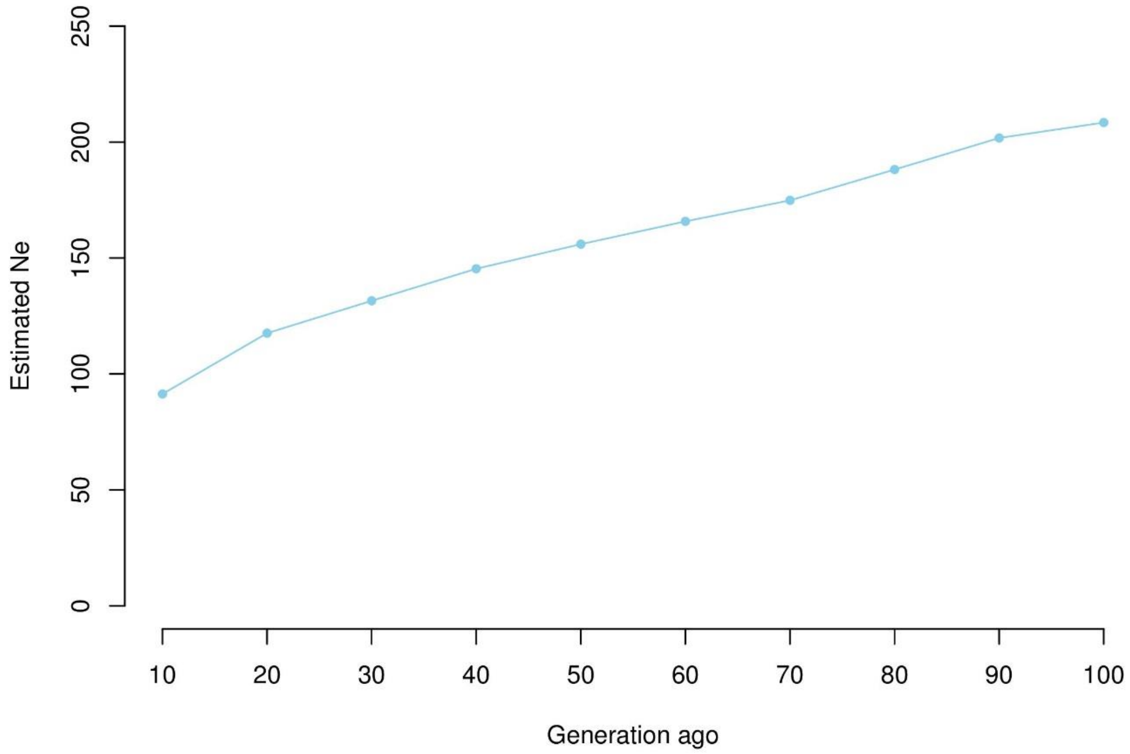

Supplementary Figure 2. Average linkage disequilibrium plotted against the median of the distance bin range (Mb) per chromosome.

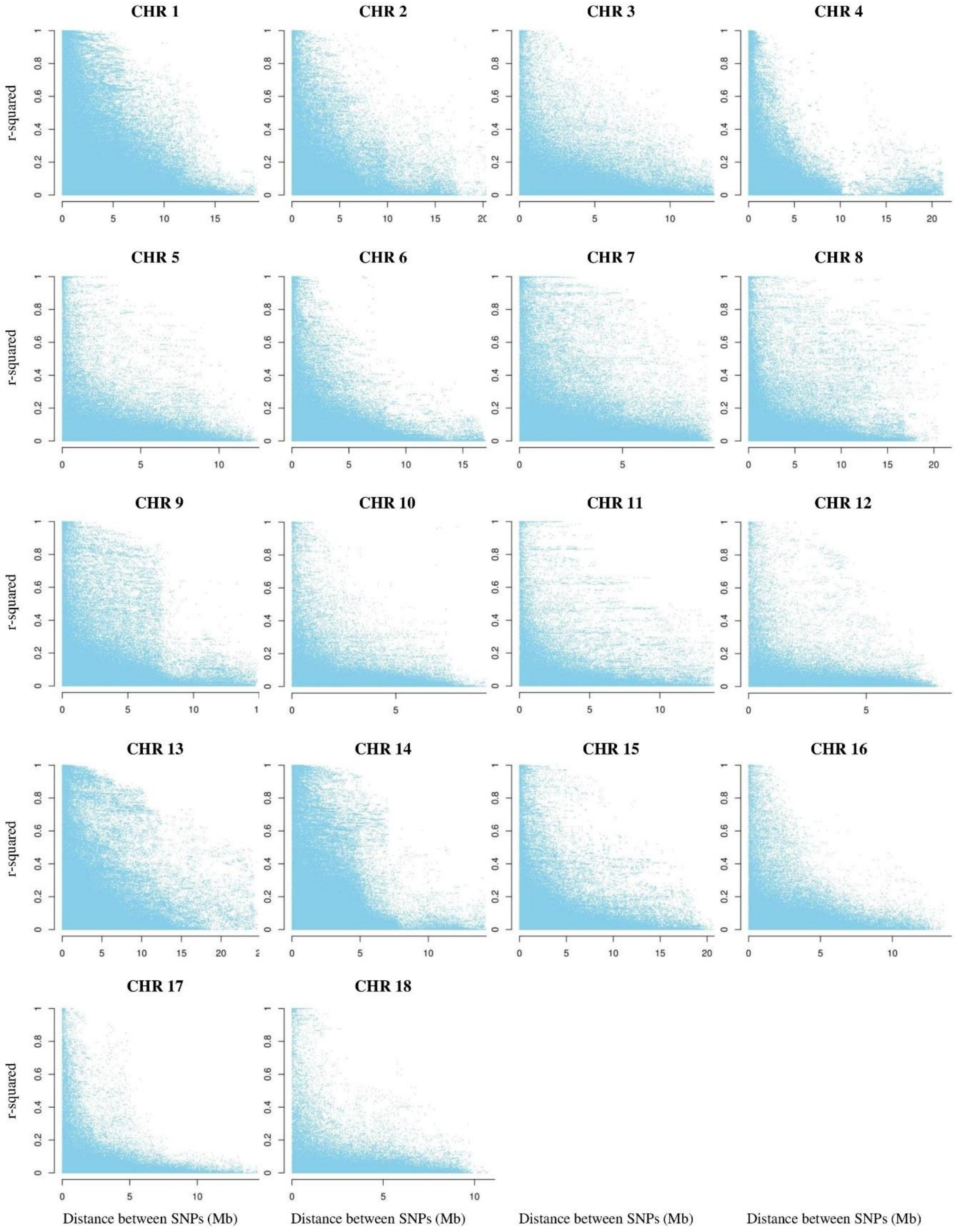

Supplementary Figure 3. Approximately purebred pig (Yorkshire) production system in each Korean GGP (Grand-Grand-parent) Farms

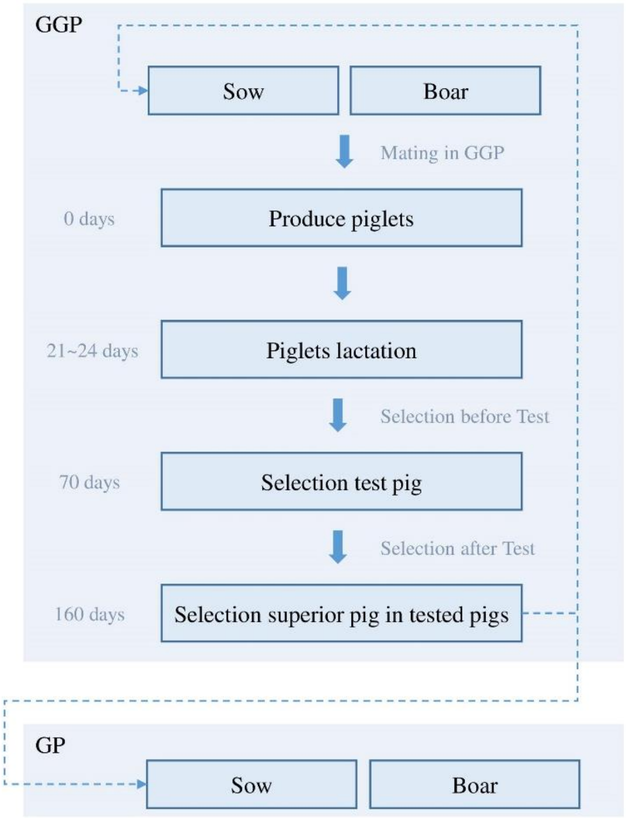

Supplementary Figure 4. Histogram of distance between SNP pairs for estimating linkage disequilibrium

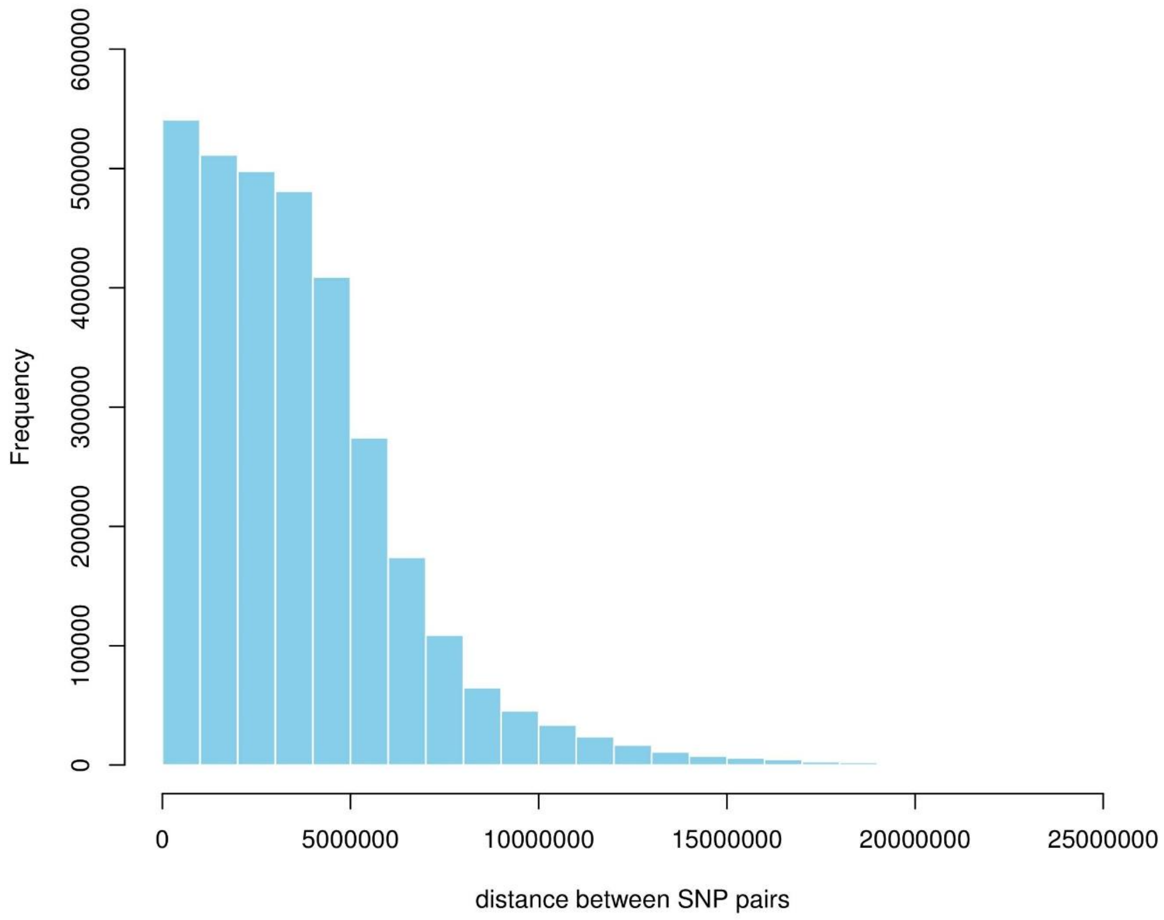

Supplement: Supplementary file 1 [file ajas-31-12-1843-supplementary.pdf]
